# P-45. Reduction of Blood Culture and Contamination During Supply Crisis of 2024: An Ecological Analysis

**DOI:** 10.1093/ofid/ofaf695.274

**Published:** 2026-01-11

**Authors:** Matias C Salomão, Kevin Querino, Alexandre M Fontes, Flavia Helena da Silva, Karin de Mello Macedo, Paola Cappellano

**Affiliations:** Grupo Fleury, São Paulo, Sao Paulo, Brazil; Grupo Fleury, São Paulo, Sao Paulo, Brazil; Grupo Fleury, São Paulo, Sao Paulo, Brazil; Grupo Fleury, São Paulo, Sao Paulo, Brazil; Grupo Fleury, São Paulo, Sao Paulo, Brazil; Grupo Fleury, São Paulo, Sao Paulo, Brazil

## Abstract

**Background:**

In mid-2024, a global shortage of blood culture (BC) bottles prompted urgent stewardship actions. In response, laboratories and hospitals launched educational campaigns to reduce unnecessary BC requests and optimize diagnostic use.

**Figure 1.**
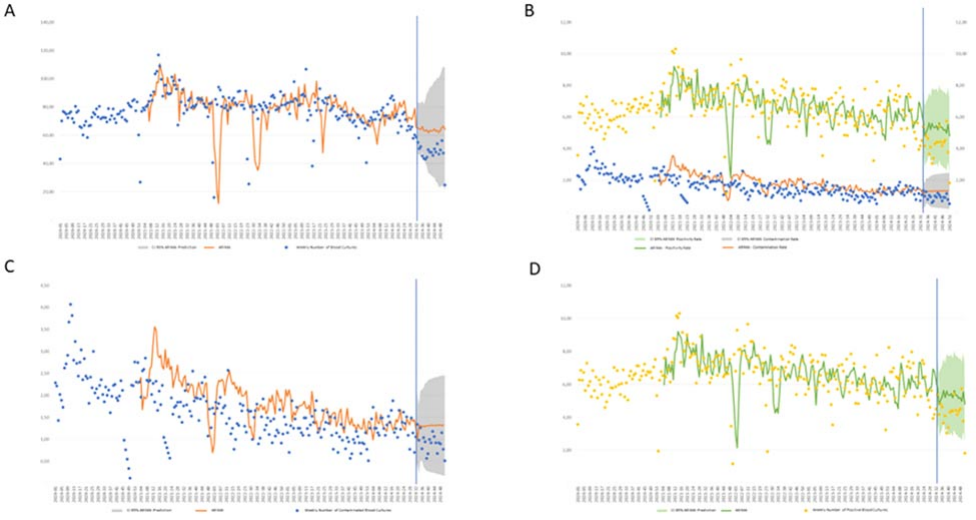
Weekly Trends in Volume, Contamination, and Positivity Rates (2020–2024) with ARIMA Forecasts after the Blood Culture Supply Crisis (week 33 - 2024).

**Methods:**

We performed an ecological time-series analysis using anonymized data from a large private laboratory in Brazil (Jan 2020–Dec 2024). The intervention (Aug–Oct 2024) included structured materials for clinicians, emphasizing evidence-based BC use, discouraging low-yield indications (e.g., pneumonia, urinary tract infection, skin/soft tissue infections without sepsis) and repeated sampling. Weekly trends were analyzed before (week 32 of 2024) and post-intervention (from week 33-52 of 2024). Outcomes included weekly BC volume, positivity and contamination rates (per standard definitions), and true-positive BCs. Interrupted time-series analysis was conducted using ARIMA in Python. We used Mann-Whitney U or paired T-test, when adequate, to compare the results found with the ARIMA´s forecast. We considered a p< 0.05 as statistically significant.

**Results:**

Among 936,981 patients (50,349 post-intervention), the weekly mean BC orders dropped from 77.9 to 49.9 post-intervention (p< 0.01). Contamination and positivity rates remained stable during the study period. However, weekly contaminated BC and positive BCs numbers decreased, respectively, from 1,9 to 0,9 and 6.6 to 4.2 post-intervention (p< 0.01), demonstrating a reduction in contamination, but also suggesting possible underdetection of bloodstream infections.

**Conclusion:**

A targeted, low-cost educational intervention during a BC bottle shortage effectively reduced test volume and contamination. However, the drop in weekly positive BCs may reflect missed bloodstream infections. While stewardship efforts improve diagnostic efficiency, they must be balanced with adequate testing to ensure patient safety. Ongoing surveillance throughout 2025 will be essential to determine whether the trends observed after the intervention are sustained over time.

**Disclosures:**

All Authors: No reported disclosures

